# Longevity and Innovation: Cardiac Resynchronization Therapy in a 100‐Year‐Old Patient With Severe Heart Failure—A 4‐Year Follow‐Up

**DOI:** 10.1155/cric/7961581

**Published:** 2026-02-11

**Authors:** Ayman Helal, Ibrahim Antoun, Rachana Prasad

**Affiliations:** ^1^ Department of Cardiology, Kettering General Hospital, University Hospitals of Northamptonshire, Kettering, Northamptonshire, UK, nhs.uk; ^2^ Department of Cardiovascular Science, University of Leicester, Leicester, Leicestershire, UK, le.ac.uk

**Keywords:** 100-year-old patient, 4-year follow-up, cardiac resynchronization therapy (CRT), severe heart failure

## Abstract

Cardiac resynchronization therapy (CRT) has been shown to improve outcomes in patients with severe left ventricular systolic dysfunction (LVSD) and electrical dyssynchrony. This case report presents a 100‐year‐old male with a history of atrial fibrillation (AF), left bundle branch block (LBBB), and ischemic heart disease, who was admitted with chest pain and episodes of bradycardia. After careful consideration, CRT‐P was successfully implanted. The patient′s condition was closely monitored postimplantation, and a 4‐year follow‐up revealed significant symptomatic improvement and functional independence. This report highlights the importance of CRT‐P in improving quality of life and managing heart failure in very elderly patients.

## 1. Introduction

Cardiac resynchronization therapy (CRT) is a well‐established treatment for patients with heart failure and electrical dyssynchrony, particularly left bundle branch block (LBBB). CRT improves left ventricular (LV) function, reduces heart failure symptoms, and enhances survival in patients with heart failure with reduced systolic function and electrical dyssynchrony. In elderly patients with multiple comorbidities, the decision to implant a CRT device must be carefully weighed against the patient′s overall prognosis, quality of life, and personal preferences [1].

This report presents a 100‐year‐old male with severe LVSD, ischemic heart disease, and AF, who underwent in‐patient CRT‐P implantation following episodes of bradycardia and intermittent AV block. We discuss the clinical decision‐making process, procedure outcomes, and the patient′s 3‐year follow‐up, illustrating the potential benefits of CRT‐P in the very elderly population.

## 2. Case Presentation

A 100‐year‐old male was admitted to the hospital after presenting with exertional chest pain. His past medical history was significant for non‐ST elevation myocardial infarction (NSTEMI), severe LV systolic dysfunction with an ejection fraction (EF) of 30%, paroxysmal AF, gout, glaucoma, and Type 2 diabetes (diet‐controlled). He had quit smoking 40 years ago and had no history of alcohol use.

On admission, his vital signs were as follows: heart rate 47 bpm, blood pressure 110/50 mmHg, respiratory rate 16 breaths per minute, and oxygen saturation 98% on room air. His neck veins were not congested, but bilateral basal crepitations were present on chest auscultation. There was no lower limb edema. Cardiac examination revealed no additional heart sounds or murmurs.

**Figure 1:**
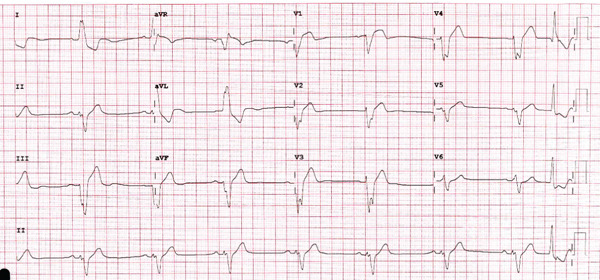
The patient′s on‐admission 12‐lead ECG.

**Figure 2:**
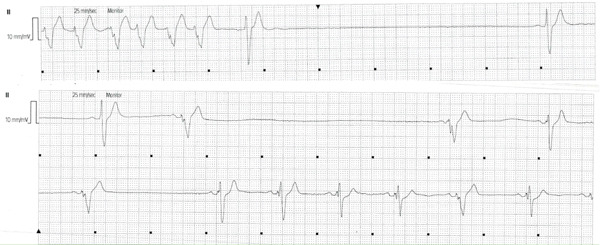
The patient had pauses on heart monitoring.

Electrocardiogram (ECG) showed sinus bradycardia and LBBB (Figure 1). Serial troponin measurements were elevated, with initial values of 46.6 and 106 ng/L, indicating an acute coronary syndrome, which was treated medically after discussion with the patient and the family. During the hospital stay, the patient had frequent episodes of pauses (Figure 2). After discussions with the patient and his family, the decision was made to implant a CRT‐P device due to recurrent bradycardia and the patient′s history of severe LVSD with anticipation of more than 40% pacing.

**Figure 3:**
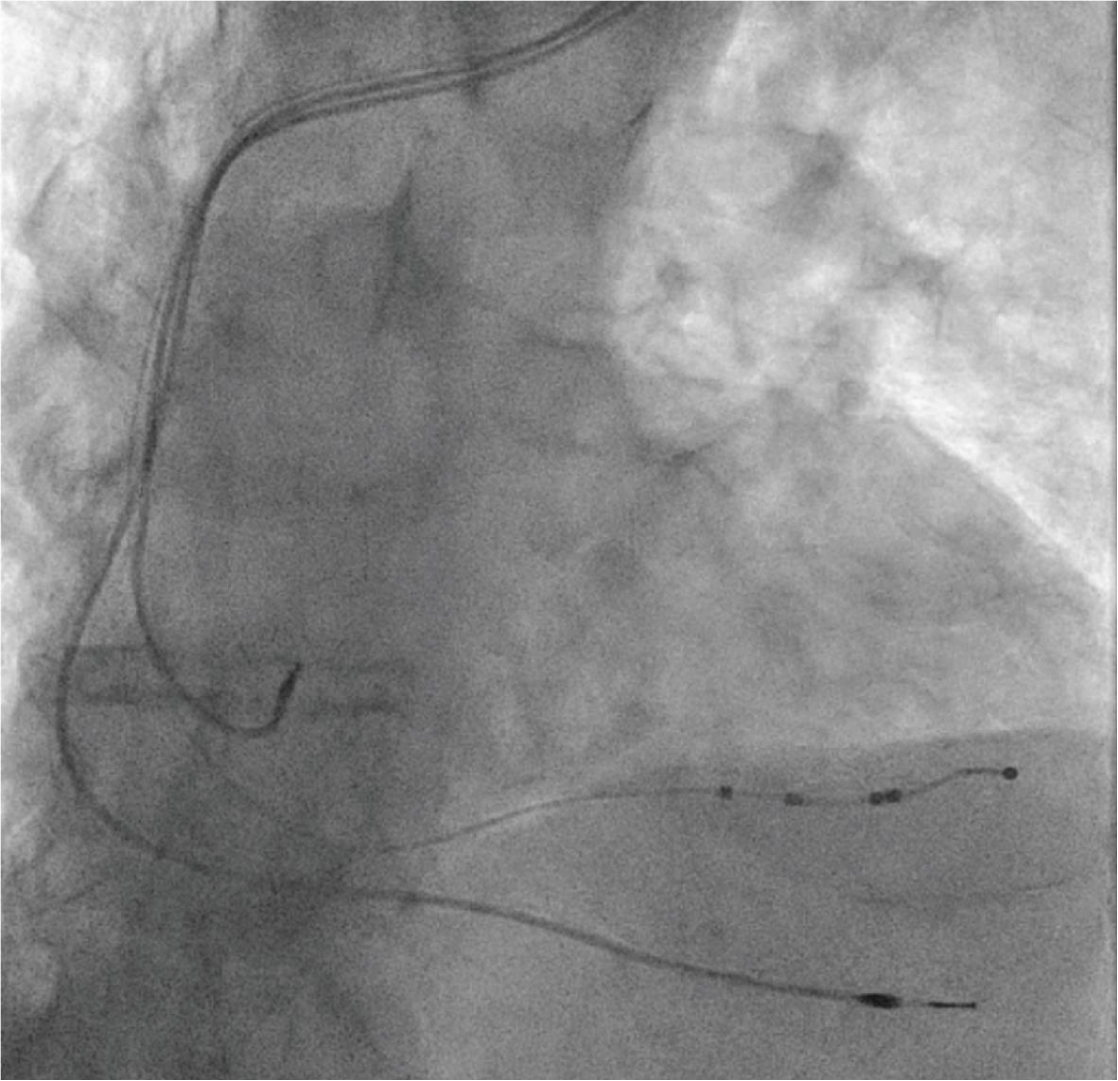
CRT‐P wires in place.

The patient underwent an implantation of an IP CRT‐P pacemaker under sterile conditions (Figure 3). Skin was prepared using ChloraPrep, and 1% lidocaine was administered for local anesthesia. Left cephalic vein cut‐down technique was used to minimize complications. The right ventricular lead (Biotronik, Solia T60, Bipolar) was placed at the RV apex with passive fixation. For the LV lead, the coronary sinus was cannulated using an extended hook catheter (Medtronic). A balloon venogram revealed a sizable posterolateral vein. An angioplasty wire was guided into the posterolateral vein, and the Attain Stability lead (Medtronic, Attain Stability Quad MRI SureScan 4798‐88, Quadripolar) was advanced. The atrial lead (Biotronik, Solia JT 53, Bipolar) was positioned in the right atrial appendage with passive fixation. Pacing, sensing, and stability tests were satisfactory. The leads were secured with Mersilk, and the pulse generator (Biotronik, Enitra 8 HF‐T QP) was positioned in a prepectoral pocket. A Tyrx absorbable antibacterial envelope was used. The pocket and wound were closed in two layers with Polysorb, and the final wound closure was completed with dissolvable Vicryl sutures.

The patient was monitored postprocedure; apixaban was restarted after 2 days. The patient′s condition stabilized, and he was discharged with secondary prevention medications and regular follow‐up arranged as outpatient.

Four years postimplantation, at the age of 104, the patient remained in good health. He was living independently, using a walking frame for mobility, and reported no significant heart failure symptoms, such as exertional shortness of breath, peripheral edema, or orthopnea. His functional status was stable, and he continued to manage daily activities, including dressing and personal hygiene, with minimal assistance. He had not experienced any device‐related complications.

## 3. Discussion

CRT‐P implantation is widely used to improve morbidity and mortality in patients with heart failure and electrical dyssynchrony. In elderly patients, especially those over 85 years old, the decision to implant a CRT device must balance the potential benefits against procedural risks and the patient′s life expectancy [1]. However, advanced age alone should not be a contraindication to CRT, as demonstrated by this case.

This case highlights several important considerations:

1. Efficacy of CRT in the elderly: Several studies have shown that CRT can improve both survival and quality of life in elderly patients, even those over 80 years old. A study of 574 patients (median age 76) receiving CRT found that older patients (≥ 80 years) had more comorbidities and were less likely to receive comprehensive medical therapy or CRT‐defibrillators. However, complications were rare, and age did not affect the symptomatic or echocardiographic response to CRT. Heart failure hospitalization rates were similar across age groups, though long‐term survival was lower for older patients. Despite these differences, CRT was equally effective in improving symptoms and LV function in older patients, supporting its use in the aging population. However, the oldest patients in this study were 87 years old [2]. Increasingly, cardiologists are treating patients with very advanced age. Frailty of age is often considered a barrier to safe invasive management. Here, we describe successful cardiac resynchronization therapy in a 100‐year‐old patient with heart failure with reduced EF secondary to ischemic cardiomyopathy and bradyarrhythmia.
2. Cephalic vein access to minimize complications: The cephalic vein cut‐down technique is a useful approach during CRT implantation to minimize complications. This technique involves a small incision to directly access the cephalic vein, allowing for the insertion of leads without the need for subclavian or axillary vein punctures, which are associated with a higher risk of complications such as pneumothorax and vascular‐related complications. By avoiding needle puncture techniques, the cephalic vein cut‐down reduces the likelihood of lead displacement, vascular injury, and other procedural risks. Additionally, this approach is particularly advantageous in elderly patients or those with complex vascular anatomy, as it provides a safer and more controlled method for lead placement, contributing to improved procedural outcomes and lower complication rates in CRT implantation [3, 4].
3. Management of bradycardia in paroxysmal AF with LBBB: In patients with AF and LBBB, bradycardia may complicate the management of heart failure. CRT‐P can address the bradycardia while improving LV synchrony. CRT devices reduce the incidence of adverse events related to bradycardia and LV dyssynchrony, thus improving overall cardiac function [5]. This was clearly demonstrated in our case.
4. Long‐term outcomes and quality of life: Elderly patients are often concerned with quality of life and maintaining independence. Follow‐up studies have shown that CRT‐P can provide durable benefits in terms of symptom control and functional capacity [6]. In this case, CRT‐P allowed the patient to maintain his independence and improve heart failure symptoms, underscoring the importance of CRT in enhancing quality of life.
5. Clinical decision‐making: The decision to proceed with CRT implantation was made after a thorough discussion with the patient and his family, considering his goals of care and preferences. Shared decision‐making is crucial in elderly patients, particularly those with multiple comorbidities, to ensure that interventions align with the patient′s values and long‐term objectives.
6. Choice of the CRT device type: A study by Mullens et al. focused on octogenarians receiving CRT, showing that while these patients experienced improvements in heart function and symptoms similar to younger individuals, most deaths were noncardiac, and arrhythmic deaths were rare. This suggests CRT‐P may be more appropriate than CRT‐D in older adults [7]. A retrospective study of 170 elderly heart failure patients (age ≥ 75) who received CRT over 10 years found no significant difference in mortality or cardiac hospitalization between those who received CRT with a defibrillator (CRT‐D) and those with a pacemaker (CRT‐P). Although CRT‐P patients had a higher burden of comorbidities, survival rates were similar between the two groups. Secondary prevention CRT‐D patients had a higher risk of hospitalization, but overall, there was no difference in hospitalization rates between CRT‐D and CRT‐P groups [8].

## 4. Conclusion

This case demonstrates the feasibility and benefits of CRT‐P implantation in a centenarian with heart failure and electrical dyssynchrony. The 4‐year follow‐up highlights the long‐term improvements in symptoms, functional capacity, and overall quality of life. While advanced age presents additional considerations in procedural decision‐making, this case suggests that CRT‐P remains a viable option for selected elderly patients, offering significant clinical benefits.

## Funding

The authors have nothing to report.

## Ethics Statement

The authors have nothing to report.

## Consent

Patient consent statement was obtained.

## Conflicts of Interest

The authors declare no conflicts of interest.

## Data Availability

Data are available on request from the authors.
